# A Cross-Cultural Analysis of Psychotic Experiences in Young Adults Across the Arab World

**DOI:** 10.1192/j.eurpsy.2026.10474

**Published:** 2026-07-31

**Authors:** L. S. Chaibi, M. Khamassi, W. Cherif, M. Bouchendira, F. Fekih-Romdhane, M. Cheour

**Affiliations:** 1 Faculty of Medecine of Tunis; 2 Psychiatry; 3 Ibn Omran, Razi Hospital, Tunis, Tunisia

## Abstract

**Introduction:**

Psychotic experiences (PEs) exist on a continuum within the general population and are influenced by a variety of sociocultural factors. While previous research has highlighted cultural variations in the expression and reporting of PEs, few studies have examined these differences within the Arab world.

**Objectives:**

This study aims to explore the cultural variations in PEs across twelve Arab countries and identify associated sociodemographic factors.

**Methods:**

A cross-sectional online survey was conducted from February to April 2024, involving 7,646 young adults (aged 18–35) from Algeria, Bahrain, Egypt, Iraq, Jordan, Kuwait, Lebanon, Morocco, Oman, Palestine, Saudi Arabia, and Tunisia. Participants completed the Arabic version of the Prodromal Questionnaire-Brief (PQ-B) to assess PEs. Sociodemographic data including country of origin, sex, marital status, education level, and lifestyle factors were also collected. Statistical analyses included bivariate comparisons and correlation analyses to examine cultural and demographic correlates of PEs.

**Results:**

Significant variations in PQ-B scores were observed across countries (p < .001). Participants from Jordan and Morocco reported the highest mean PQ-B scores (31.56 ± 21.54 and 34.17 ± 22.17, respectively), while those from Tunisia and Kuwait reported the lowest (25.27 ± 20.75 and 26.09 ± 18.92, respectively). Higher PEs were significantly associated with female sex (p = .043), single marital status (p < .001) and lower educational attainment (p = .026). No significant associations were found with alcohol or cannabis use.

**Conclusions:**

This study confirms significant cultural variation in psychotic experiences among young adults across Arab nations, influenced by sociodemographic factors. These findings highlight the necessity of culturally adapted mental health approaches and early intervention strategies tailored to regional contexts within the Arab world.

**Disclosure of Interest:**

None Declared

